# The Molecular Assembly of Amyloid Aβ Controls Its Neurotoxicity and Binding to Cellular Proteins

**DOI:** 10.1371/journal.pone.0024909

**Published:** 2011-09-23

**Authors:** Claudia Manzoni, Laura Colombo, Paolo Bigini, Valentina Diana, Alfredo Cagnotto, Massimo Messa, Monica Lupi, Valentina Bonetto, Mauro Pignataro, Cristina Airoldi, Erika Sironi, Alun Williams, Mario Salmona

**Affiliations:** 1 Department of Molecular Biochemistry and Pharmacology, Mario Negri Institute for Pharmacological Research, Milan, Italy; 2 Department of Oncology, Mario Negri Institute for Pharmacological Research, Milan, Italy; 3 Dulbecco Telethon Institute, Milan, Italy; 4 Department. of Biotechnology and Bioscience, University of Milano-Bicocca, Milan, Italy; 5 Department of Veterinary Medicine, University of Cambridge, Cambridge, United Kingdom; Centre National de la Recherche Scientifique - University of Bordeaux, France

## Abstract

Accumulation of β-sheet-rich peptide (Aβ) is strongly associated with Alzheimer's disease, characterized by reduction in synapse density, structural alterations of dendritic spines, modification of synaptic protein expression, loss of long-term potentiation and neuronal cell death. Aβ species are potent neurotoxins, however the molecular mechanism responsible for Aβ toxicity is still unknown. Numerous mechanisms of toxicity were proposed, although there is no agreement about their relative importance in disease pathogenesis. Here, the toxicity of Aβ 1–40 and Aβ 1–42 monomers, oligomers or fibrils, was evaluated using the N2a cell line. A structure-function relationship between peptide aggregation state and toxic properties was established. Moreover, we demonstrated that Aβ toxic species cross the plasma membrane, accumulate in cells and bind to a variety of internal proteins, especially on the cytoskeleton and in the endoplasmatic reticulum (ER). Based on these data we suggest that numerous proteins act as Aβ receptors in N2a cells, triggering a multi factorial toxicity.

## Introduction

Alzheimer's disease is the most widespread form of dementia worldwide. Characteristic pathological lesions are senile amyloid plaques, vascular amyloidosis and neurofibrillary tangles. The amyloid aggregates are formed by Aβ peptides of various amino-acid lengths [1] derived from the processing of a membrane protein (amyloid precursor protein, APP). The most abundant peptides are Aβ 1–40 and Aβ 1–42, the first being the prevalent fragment, the second the most amyloidogenic. Numerous Aβ species differing in their aggregation state have been isolated or produced [2]–[5]. Aβ species are active neurotoxins and it is possible that not only one single Aβ assembly is responsible of the neurodegeneration, but probably the complexity of Alzheimer's disease requires numerous active Aβ species to be considered, all with the same amino acid composition but with different aggregation state and 3D structure. One of the major challenges in deciphering the pathogenesis of Alzheimer's disease is to clarify the mechanisms whereby these species lead to neuronal loss. An abundance of different molecular alterations have been described in cells lines and cultures following Aβ exposure. Despite significant efforts by many groups, there is still no consensus on the relative importance of these different molecular events and there is no clear, unique, causative pathway [6]. Several papers have suggested that there may be a single, specific, “death receptor” accounting for Aβ-induced toxicity, but, to date, at least 9 different proteins have been described for that role without reaching any general agreement. In this study different Aβ 1–40 and Aβ 1–42 molecular assemblies were investigated to clarify their mechanism of toxicity. The data obtained showed that toxicity in the N2a cell model depended on Aβ peptide aggregation states. When toxic, Aβ peptides had a high tendency to cross the plasma membrane and bind to multiple proteins, especially those associated with membrane compartments and the cytoskeleton. In consequence, we propose that Aβ peptides can induce cell toxicity by binding to a variety of proteins leading to the activation of multiple pathways that can generate different, apparently unrelated, toxic downstream events within the cell. This model for Aβ toxicity does not require the existence of one, single, specific Aβ receptor.

## Results

### 2.1. Cellular localization of Aβ 1–42 toxic oligomers

**Figure 1:**
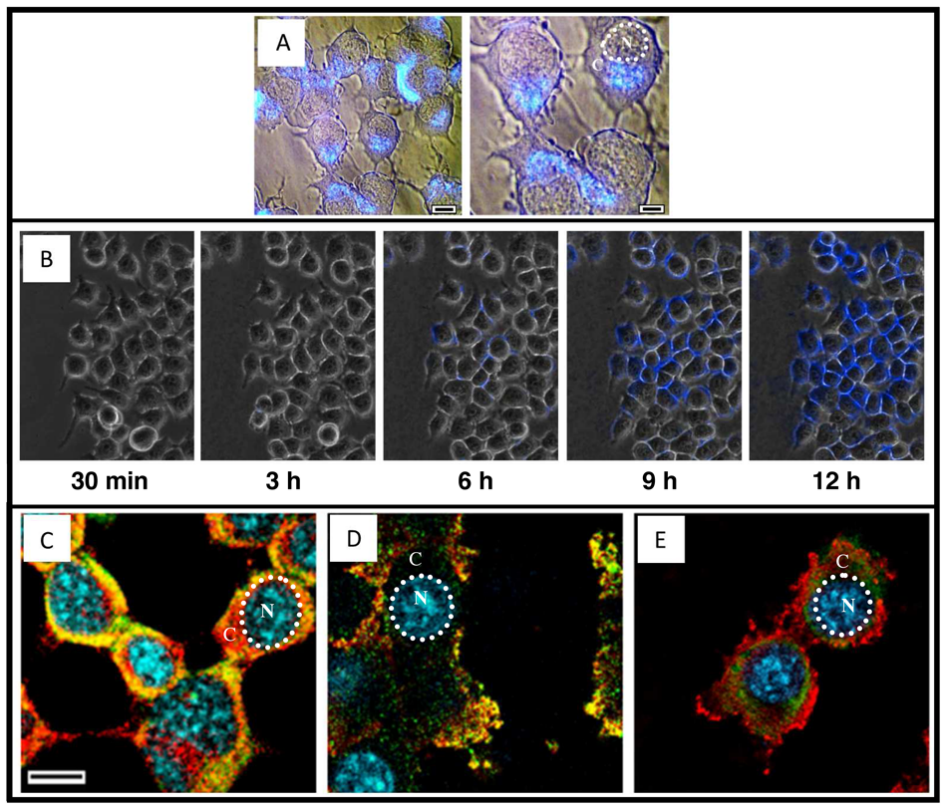
Aβ 1–42 oligomer distribution in N2a cells. A) High magnified, fluorescence microscopy pictures showing the cellular localization of fluorescent dye 6 hours after 30 µM Aβ 1–42-EDANS large oligomers incubation. The fluorescence is mainly confined to the cytoplasm [C] and did not penetrate into the nucleus [N]. Scale bar 10 and 5 µm. B). The kinetic of accumulation was carried out by time lapse recording experiments coupled to fluorescence microscope acquisition (40× of magnification). Each single image represents the merge between the contrast phase signal and the fluorescence of the field excited in the UV range (from 380 to 425 nm of wavelength), Scale bar 40 µm. C-D-E) N2a cells were treated with 30 µM Aβ 1–42 large oligomers for 6 hours prior to immunocytochemistry analysis. C) vimentin, D) GRP-78 and E) cathepsin D (all FITC) plus Aβ 6E10 staining (TRIC 546); nuclei were stained with Hoechst 33285. Scale bar. 15 µm. Images were merged by superimposing single fluorescence images.

Oligomers were produced and characterized in detail (chemico-physical and toxicological characterization) with both untagged and EDANS (ethyldiaminonaphthalene-1-sulfonic acid)-tagged Aβ 1–42 peptides (Figure S1 and Supporting Information S1) following procedures previously validated and published [7], [8]. N2a cells were treated with Aβ 1–42-EDANS toxic oligomers (EDANS can be directly visualized in fluorescence microscopy by the use of an UV light source and a DAPI filter). No signal was visible after 5 minutes treatment (data not shown) while, 6 hours after peptide exposure, most of the fluorescence appeared to be intracellular, accumulating in the perinuclear area (unstained nuclei); with the peptide apparently located in dense, extremely bright granules (Figure 1A). Time dependent, oligomer distribution in cells was then recorded by Time-Lapse fluorescence microscopy. N2a cells were treated with Aβ 1–42-EDANS oligomers and images recorded every 15 minutes, ending 16 hours after peptide administration. Selected frames from different movies were organized in temporal sequence (Figure 1B), clearly demonstrating EDANS-peptide internalization in N2a cells.

As confirmation, peptide internalization was investigated in the absence of the EDANS fluorophore after treatment of N2a cells with toxic Aβ 1–42 untagged oligomers. In this second experimental setting, Aβ 1–42 detection was performed by anti-Aβ 6E10 antibody (immunocytochemistry) using confocal microscopy; markers for peptide internalization were selected as follows: vimentin (cytoskeleton), cathepsin D (lysosomes), GRP-78 (endoplasmic reticulum and associated membranes) and Hoechst 33285 (nuclei). N2a cells were treated as before and after 6 hours the unlabeled peptide, detected by the 6E10 antibody, was clearly able to enter N2a cells since its fluorescence co-localized with vimentin and GRP-78 (Figure 1C and 1D). Unexpectedly, the internalized peptide did not co-localize with cathepsin D and this result was considered a negative control for the immunocytochemistry procedure (Figure 1E). Again, oligomers were not detected in nuclei.

### 2.2. Investigation of Aβ binding partners

**Figure 2:**
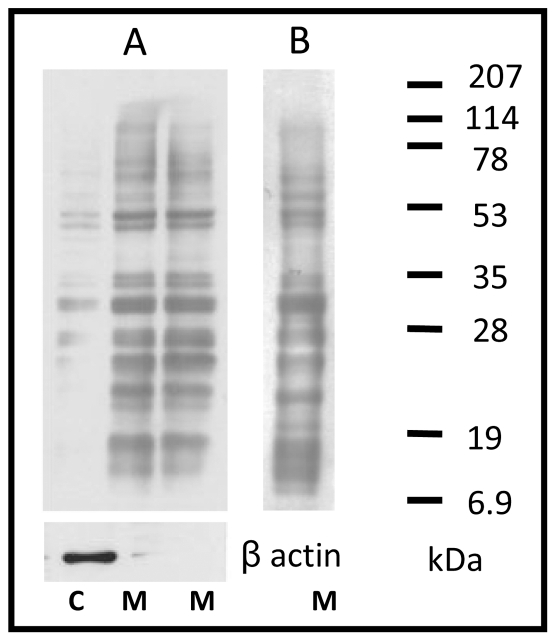
Far Western Blot with cytosolic (C) and membrane proteins (M). Fifteen µg of proteins from cytosol and membrane fractions were blotted after electrophoresis. Far Western Blot was performed with A) 100 ng/ml of untagged Aβ 1–42 enriched in oligomers. Actin is shown as a marker for the cytosolic fraction. B) 100 ng/ml of EDANS-tagged Aβ 1–42 enriched in oligomers.

Following the suggestion that Aβ toxic oligomers were able to enter N2a cells, the possibility that these particles bind to intracellular proteins was investigated by using a high-throughput approach. N2a cells were lysed using a detergent-free solution in order to preserve membrane integrity; proteins were then separated by centrifugation into two fractions: (i) cytosol; (ii) membrane fragments and organelles. The resulting fractions were blotted onto nitrocellulose after SDS-PAGE fractionation and the membrane was washed overnight in TBST to promote the maximum of protein renaturation achievable [9]–[11]. The nitrocellulose membrane was incubated for 2 hours with 100 ng/ml Aβ 1–42 toxic (EDANS-tagged or untagged) oligomers followed by staining with the 6E10 antibody. At variance with the cytosol numerous proteins in the membrane fraction were able to interact with Aβ 1–42 oligomers (Figure 2A and 2B).

A proteomic approach was then used to identify the proteins involved in Aβ binding. A particular type of 2D gel separation was selected [12]. After the second dimension separation, proteins were partially electroblotted onto nitrocellulose. The membrane was processed as described before, with exposure to 100 ng/ml Aβ 1–42 oligomers. The partially transferred gel was stained by Coomassie Instant Blue. Images of the membrane and Coomassie stained gel were superimposed to allow the identification (on the gel) of the Coomassie spots that contained proteins positive for Aβ binding (as verified on the membrane). Those spots were cut out from the gel and digested by trypsin. This procedure was applied to seven experimental replicates, to obtain a reliable read out. In four cases out of seven the digested spots were analyzed by MALDI-TOF spectrometry; in the other three cases analysis was performed by MALDI-TOF-TOF spectrometry to obtain confirmation of the results. The generated peak lists were used to search the SwissProt database using the MASCOT searching algorithm (Table 1). Many different proteins were identified, principally coming from cytoskeleton and intracellular membrane compartments. This, again, suggested the possibility for the peptide to be internalized and to generically bind to a set of different proteins and not to only one specific binding partner.

**Table 1:** Identification of membrane proteins able to bind Aβ 1–42 large oligomers.

Identified Protein	Symbol	Uniprot-Swiss-	MW	Score	Cov %	# Matches	Score	Cov %	# Matches
		Prot#	(kDa)	(MS/MS)	(MS/MS)	(MS/MS)	(MS)	(MS)	(MS)
60S ribosomal protein L15	RL15_MOUSE	Q9CZM2	24.1	66	64	21	115	63	16
60S ribosomal protein L7a	RL7A_MOUSE	P12970	30.1	-	-	-	66	25	8
Heterogeneous nuclear ribonucleoproteins A2/B1	ROA2_MOUSE	O88569	37.4	67	61	25	62	32	12
Heterogeneous nuclear ribonucleoproteins A1	ROA1_MOUSE	P49312	34.3	-	-	-	66	40	10
Vimentin	VIME_MOUSE	P20152	53.6	257	70	56	257	66	41
Peripherin	PERI_MOUSE	P15331	54.2	149	70	44	75	41	19
T-complex protein 1 alpha B	TCPA2_MOUSE	P11983	60.4	81	49	36	58	23	13
Heat shock cognate 71 kDa protein	HSP7C_MOUSE	P63017	70.8	168	48	35	91	33	19
Heat shock-related 70 kDa protein 2	HSP72_MOUSE	P17156	69.7	94	34	23	-	-	-
Nucleolin	NUCL_MOUSE	P09405	76.7	113	38	40	99	35	35
Polyadenylate-binding protein 1	PABP1_MOUSE	P29341	70.6	145	59	59	104	45	27
Probable ATP-dependent RNA helicase	DDX5_MOUSE	Q61656	69.3	72	47	46	-	-	-
Ketatin, type II cytoskeletal 1b	K2C1B_MOUSE	Q6IFZ6	61.3	57	43	32	-	-	-
Elongation factor 1-alpha 1	EF1A1_MOUSE	P10126	50.1	86	28	17	-	-	-
60S ribosomal protein L3	RL3_MOUSE	P27659	46.1	103	56	39	-	-	-
Eukaryotic translation initiation factor 3 subunit A	EIF3A_MOUSE	P23116	161.8	197	54	94	-	-	-
Eukaryotic translation initiation factor 5B	IF2P_MOUSE	Q05D44	137.5	58	37	57	-	-	-
Eukaryotic translation initiation factor 3 subunit L	EIF3L_MOUSE	Q8QZY1	66.570	91	38	28	-	-	-
Isoleucyl-tRNA synthetase, cytoplasmic	SYIC_MOUSE	Q8BU330	144.2	116	47	71	-	-	-
Valyl-tRNA synthetase	SYVC_MOUSE	Q9Z1Q9	140.1	68	34	55	-	-	-
Trypeptidyl-peptidase 2	TPP2_MOUSE	Q64514	139.8	58	30	53	-	-	-

Score: the protein score was calculated by the MASCOT algorithm. In the case of MS/MS identifications, the score is the combined between MS and MS/MS analyses. The identification was considered reliable when the given score for the single protein was above 55; ppm was always under 50. Cov%: sequence coverage.

### 2.3. Aβ 1–40 and Aβ 1–42 peptides

In the set of experiments reported until now, only toxic oligomers from Aβ 1–42 peptides have been used. However, from the literature it is known that other aggregation forms of Aβ 1–42 as well as Aβ 1–40 peptides are implicated in the Alzheimer's pathology; all of them having different impact on neuronal toxicity [13]. Aβ 1–40 and 1–42 peptide samples differently enriched in amyloid assemblies to encompass the amyloidogenic process from the monomer to the mature fibril (monomers – oligomers - fibrils) were prepared and characterized (Figure S2, Table S1 and Supporting Information S1) with the aim to verify if different toxicological profiles, typical of different Aβ aggregative forms, may hide different protein-binding abilities.

**Figure 3:**
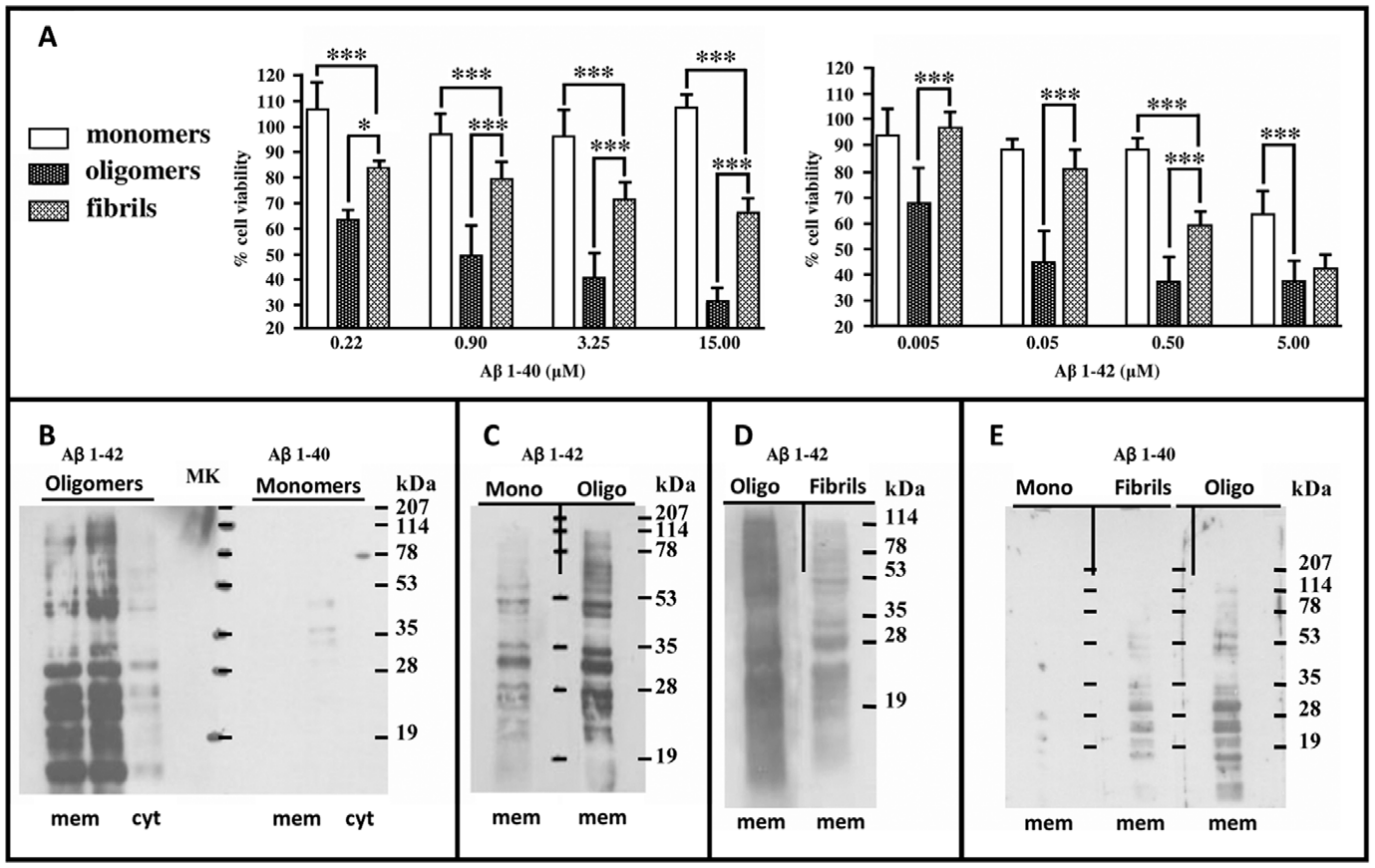
Aβ toxicity and Far Western Blot assays. A) N2a cell viability after treatment with Aβ 1–40 species or Aβ 1–42 species, concentration range from 0.22 µM to 10 µM and 50 nM to 5 µM respectively. ANOVA followed by Bonferroni's post hoc test; *** p<0.001; * p<0.05; p>0.05 not significant (ns). Mean and SD; N = 3 or more different experiments, 4 replicates each. B-C-D-E) Comparisons of binding capacities among different peptide preparations. Fifteen µg proteins from cytosolic (cyt) and membrane fractions (mem) were run on SDS-PAGE and blotted onto nitrocellulose. Far Western Blot was performed with B 1) 100 ng/ml Aβ 1–42 oligomers; B 2) 100 ng/ml Aβ 1–40 monomers; C 1) 100 ng/ml Aβ 1–42 monomers; C 2) 100 ng/ml Aβ 1–42 oligomers; D1) 100 ng/ml Aβ 1–42 oligomers; D2) 100 ng/ml Aβ 1–42 fibrils; E1) 1 µg/ml Aβ 1–40 monomers; E2) 1 µg/ml Aβ 1–40 fibrils; E3) 1 µg/ml Aβ 1–40 oligomers. Membranes were incubated with peptide solutions separately; then pooled to be stained concurrently with the same antibody incubations and the same film exposure.

Different Aβ preparations were assessed for their ability to alter N2a cells viability (Figure 3A). N2a cells were incubated with peptide preparations for 72 hours (concentration range from 5 nM up to 15 µM) and cell damage was evaluated by the MTT (3-(4,5-Dimethylthiazol-2-yl)-2,5-diphenyltetrazolium bromide) reduction assay. The cell impairment followed a dose-response pattern. As expected from literature, the most toxic Aβ 1–42 preparation was the one enriched in oligomers. Monomers and preparations enriched in fibrils were less active over the same range of peptide concentrations; in particular monomers produced no significant change in MTT with the exception of the higher dosage; Aβ 1–42 fibrils partially retained a toxic activity, however less than in comparison with the preparation of oligomers. Interestingly, a similar trend was observed with Aβ 1–40 assemblies as well. As expected, Aβ 1–42 showed a stronger toxic effect in comparison with Aβ 1–40, being Aβ 1–42 toxic species active at very low concentrations in comparison with Aβ 1–40. The difference was especially remarkable in the comparison between the two monomeric preparations. In general monomers did not display the cytotoxicity observed for oligomers and fibrils, nevertheless in the case of Aβ 1–42 the monomer showed a partial toxicity at the higher peptide concentration (5 µM). This was not recorded for Aβ 1–40 for which the monomer was still not active even at 15 µM peptide dosage.

As the six Aβ 1–40 and Aβ 1–42 preparations differed in their ability to impair N2a cell viability, they were examined for their binding ability for proteins from N2a cells. Membrane proteins from N2a cells were blotted onto nitrocellulose after SDS-PAGE. The nitrocellulose membranes were cut in two parts and processed separately with solutions containing different Aβ conformations. After peptide exposure, the membranes were reunited, to be equally probed with antibodies and uniformly processed during film exposure to obtain the final image. Comparisons made were between: Aβ 1–42 oligomers *vs* Aβ 1–40 monomers (Figure 3B); Aβ 1–42 monomers *vs* Aβ 1–42 oligomers (Figure 3C); Aβ 1–42 fibrils *vs* Aβ 1–42 oligomers (Figure 3D); Aβ 1–40 monomers, Aβ 1–40 oligomers *vs* Aβ 1–40 fibrils (Figure 3E). In all these comparisons there was a direct correlation between the toxicity of the peptide preparations and their ability to bind proteins: the more toxic the peptide, the greater the intensity of binding to cell proteins especially in the membrane fraction.

## Discussion

Numerous and apparently unrelated molecular alterations have been associated with Aβ toxicity. Drawing these various data together to generate an exhaustive description of the mechanism of Aβ-induced neuronal toxicity has proved difficult. Recently, the existence of one receptor able to interact with Aβ peptides triggering the toxic response in neurons has been proposed [14]. However, this result is still quite controversial [15] because more than one single and specific protein seem to be involved in triggering of Aβ dependent neuronal alterations. Multiple Aβ binding candidates have been described (e.g. α7 nicotinic acetylcholine receptor [16], RAGE (Receptor for Advanced Glycosylation End-products) [17], Aβ precursor protein [18], NMDA receptor, P75 receptor (neurotrophin receptor P75NTR) [19], scavenger receptors [20], CD36 [21], and low density receptor-related lipoproteins [22]). Overall, these data suggest that Aβ assemblies are toxins that interact with a variety of heterogeneous partners instead of a single one. This was also the outcome of a recent paper by Olzscha that showed that oligomers of artificial amyloidogenic peptides, as well as Aβ species, bind in the cells to a wide number of pre-existent and newly synthesized proteins simply on the basis of specific sequence features [23].

In this paper we show that Aβ 1–42 oligomers covalently bound to EDANS were able to enter N2a cells and accumulate in the perinuclear area. Peptide internalization in cells was confirmed by antibody tracking of untagged, Aβ toxic oligomers and colocalization studies with markers for cytoskeleton, ER, lysosomes and nuclei. These results suggested that Aβ toxic oligomers cross plasma membrane and bind the intracellular organelles and cytoskeleton. Far Western blot analysis was used to investigate Aβ interacting partners since this technique has been used in the past to investigate the presence of proteins in brain extracts able to bind synthetic Aβ derived diffusible ligands (ADDLs) [24], identify the shortest segment in Aβ peptides responsible for interaction with proteins [25], or identify the interaction partners of the cellular form of prion protein PrP^c^ [26]. Notably, Aβ 1–42 toxic oligomers bound to many proteins of the membrane fraction (i.e. containing intracellular membranes, organelles and some parts of the polymerized cytoskeleton attached to the inner surface of the plasma membrane), at variance with soluble cytosolic proteins which showed less Aβ oligomers binding. Among the numerous proteins in the membrane fraction which were identified by mass spectrometry as Aβ-interacting partners, many of them are involved in the protein synthesis (ribosomal proteins RL15, RL7a, L3; polyadenylate binding protein 1; translation initiation factor 3A, 5B, 3L; elongation factor 1 alpha1; isoleucyl and valyl-trna syntetase) as well as proteins associated with the cytoskeleton. This suggests that Aβ species can potentially interfere with key cellular functions like protein synthesis, cytoskeleton organization, m-rna processing. These results, obtained following a totally different approach, are backing-up the already mentioned data from Olzscha [23] describing the nature of interactions between β proteins and key cellular hubs involved, among different functions, in transcription, translation vesicular transport and cytoskeleton, these interactions were described to be correlated with the β aggregates cytotoxicity. Interestingly proteins involved in all these processes have been identified as well as stress response proteins to be altered in level of expression during Aβ induced cell toxicity [27] again pointing out the multiplicity of cell processes altered as a response to Aβ stimulation.

Dahlgren et al. [13], showed that Aβ 1–42 toxicity was directly associated with its molecular assembly. This was confirmed here by us using our Aβ 1–42 samples, moreover we show that the toxicity of Aβ 1–40 is secondary to peptide folding as well. For both Aβ 1–40 and 1–42, the toxicity increased with the accumulation of oligomers and protofibrils; finally, the incorporation of oligomers into mature fibrils reduced the toxicity of the preparations. A comparison of protein binding capacity and toxicity of different Aβ 1–40 and Aβ 1–42 preparations was also carried out. The protein binding capacity in the cell was directly correlated with peptide toxicity, confirming the importance of the molecular assembly, irrespectively with amino acid composition, in dictating the biological behaviour of Aβ.

In conclusion, our studies demonstrate that Aβ toxic species cross the plasma membrane and accumulate within N2a cells by docking to a variety of proteins. Therefore in our experimental conditions, our data dismiss the hypothesis of the presence of a single “death receptor” and put forward the idea that many proteins can be, under different extents, target of Aβ binding. The ability of Aβ toxic species to enter into cells and interact with many target proteins suggests the idea that a plurality of molecular alterations after massive protein binding could interfere generically, but concurrently, in numerous independent, physiological pathways. This may therefore explain the presence of a large number of molecular alterations associated with Aβ-induced toxicity in literature as well as many proposed Aβ receptors that, up to now, have always been considered independently.

## Materials and Methods

### Peptide storage and handling

Aβ 1–40 and Aβ 1–42 were prepared by solid-phase peptide synthesis [7]. Lyophilized samples were kept at −20°C until use. Peptides were never conserved in solution, they were freshly dissolved immediately before use, and residual peptide solutions were always discarded after each experiment. Production and characterization of different aggregative Aβ species (Aβ 1–40 and Aβ 1–42 monomers, oligomers and fibrils) have been carried out as described in a previous publications [7], [8]. Monomers have been produced following a disaggregation protocol based on the use of formic acid and trifluoro acetic acid. Protocols for peptide preparation are discussed in details as supplementary information and data are provided to describe the aggregation state for each of the Aβ samples. Aβ samples were prepared under sterile conditions to avoid bacterial contamination.

### EDANS fluorescence

N2a cells (commercialized by ATCC, number CCL-131, for more information web page http://www.lgcstandards-atcc.org/LGCAdvancedCatalogueSearch/ProductDescription/tabid/1068/Default.aspx?ATCCNum=CCL-131&Template=cellBiology) grown on glass coverslips were treated with 30 µM EDANS (ethyldiaminonaphthalene-1-sulfonic acid)-tagged Aβ 1–42, and incubated for 5 minutes or 6 hours. Cells were washed twice in PBS and coverglases were mounted onto glass slides using Fluor Save mounting medium (Calbiochem, San Diego, CA). Slides were allowed to dry, before analysis using an Olympus fluorescence microscope (Olympus, Hamburg, Germany) equipped with an Olympus U-RFL-T UV source and DAPI emission filter. All fluorescence images were acquired using the same exposure parameters. Merged images between bright-field and fluorescence were obtained with the Adobe Photoshop software CS 8.0 (Adobe System Incorporated).

### Time Lapse recordings

N2a cells were grown on tissue culture treated Ibidi μ-slides eight-well dishes (Ibidi, Munich, Germany). Cells were treated with 30 µM EDANS-tagged Aβ 1–42, and the wells were completely covered with mineral oil. Image recordings started 30 minutes after cell treatment using an Olympus time lapse microscope (Olympus, Hamburg, Germany) equipped with a cell incubator box, Olympus U-RFL-T UV source and DAPI emission filter. Different positions were selected and images in bright-field and in fluorescence were recorded every 15 minutes for 16 hours. Single images were used to compose movies. Acquisition and movie composition were done with the Cell∧R 3.0 software (Olympus, Hamburg, Germany).

### Immunocytochemistry

N2a cells were fixed with Carnoy's solution (1 h, room temperature) and then incubated with Hoechst 33258 dye (1 h, room temperature; 1∶200; Sigma-Aldrich). Cells were then permeabilized with 0.1% Triton X-100 with 10% foetal bovin serum and incubated overnight (4°C) with mouse primary antibodies against the Aβ 1–42 peptide (1∶500; 6E10 Signet) in PBS containing 0.1% Triton X-100 and 10% foetal bovin serum. Cells were washed and incubated for 1 hour at room temperature with the secondary anti-mouse IgG conjugated with Alexa 546 (1∶1000; Molecular Probes, Carlsbad, CA) in PBS containing 1% FBS. Cells were then washed and incubated overnight at 4°C with primary antibodies against GRP78 (goat; 1∶200; Santa Cruz Biotechnology), vimentin (goat; 1∶200; Chemicon) or cathepsin (rabbit; 1∶300; Santa Cruz) in PBS containing 0.1% Triton X-100 and 10% foetal bovin serum. After washing, cells were incubated for 1 hour at room temperature with the secondary anti-rabbit or anti-goat IgG conjugated with Alexa 488 (1∶1000; Molecular Probes, Carlsbad, CA) in PBS containing 1% FBS.

### Protein extraction from N2a cells

Mild cell lysis for protein fractionation - slightly modified method from [28], [29] - was achieved with hypotonic lysis buffer (CLBi) composed of 10 mM NaCl, 5 mM EDTA, 1 mM KH_2_PO_4_, 5 mM NaHCO_3_, complete protease inhibitor cocktail and 1 mM sodium orthovanadate in 10 mM HEPES buffer. CLBi was added directly to the flask, and cells were scraped off and collected. Cell lysis was improved by using a 26G needle. Cell debris were eliminated by centrifugation (2×10 min centrifugation at 6.6 g) and the supernatant was collected as the post-nuclear fraction. This fraction was then centrifuged for 3 hours at 14 000 rpm at 4°C (Avanti J-25 centrifuge; JA 18.1 rotor; Beckman Coulter, Fullerton, CA). The supernatant was collected as the cytosolic fraction; the pellet containing membrane/organelles was washed once and suspended in PBS containing complete protease inhibitor cocktail and 1 mM sodium orthovanadate. Protein was quantified by the BCA assay.

### Far Western Blot assay

Proteins were diluted 1∶1 with the loading buffer containing 12% (w/V) SDS and 100 mM dithiothreitol in Tris-HCl buffer 0.5 M, pH 6.8, and immediately denatured at 100°C for 5 minutes. Samples were analyzed using Tris-glycine SDS-PAGE (1.5 mm thick, 12.5% gradient gels) and blotted in a semi-dry apparatus (GE Healthcare Easton Turnpike, CT) onto nitrocellulose. The blotting membrane was blocked from 1 hour to overnight in 5% non-fat milk. After washing in TBST the membrane was incubated 2 hours with 100 ng/ml Aβ peptides in TBST and the membrane was processed as for Western Blot with the primary antibody 6E10 (1∶5000, Signet Laboratories, Dedham, MA) and anti-mouse HRP conjugated secondary antibody (1∶2000, DAKO, Carpinteria, CA) using an ECL detection kit (GE Healthcare Easton Turnpike, CT).

For the experimental control the nitrocellulose membrane was incubated only with Aβ peptide and anti-mouse secondary antibody, without the 6E10 primary antibody.

### 2D gel electrophoresis and mass spectrometry

2D gel electrophoresis was as described by Hartinger at al. [30]. The first dimension was done as polyacrylamide gel electrophoresis in the presence of urea and benzyldimethyl-n-hexadecylammonium chloride (16-BAC). Gels were then fixed in a solution containing isopropanol∶water∶acetic acid and stained with 0.15% Coomassie blue R-250. Lanes were cut and re-equilibrated in 100 mM Tris-HCl pH 6.8 1 hour before second dimension electrophoresis performed as conventional Tris-glycine SDS-PAGE (1.5 mm thick, 12.5% gels). Gels were partially electroblotted onto nitrocellulose (0.2 µm pore size, Whatmann, GE Healthcare, Easton Turnpike, CT) by a semi-dry apparatus (GE Healthcare Easton Turnpike, CT) for 35 min, using 0.8 mA/cm^2^. Blotting membranes were processed as for Far Western blot and gels were stained by Coomassie Instant blue (Expedeon, Cambridge, UK). Images from gels and membranes were acquired and superimposed to select corresponding spots in the gel.

Spots were excised and processed for mass spectrometry. Spots were destained in 40% ethanol and shrunk in acetonitrile. Proteins were then reduced for 1 hour at 37°C by 10 mM dithiothreitol and carbamidomethylated 20 min by 55 mM iodoacetamide. Proteins were digested overnight by the addition of 12.5 ng/µl sequence grade modified bovin trypsin (Roche Applied Science, Indianapolis, IN). Trypsin digestion was blocked by addition of 0.1% TFA and peptides were spotted onto MALDI-TOF/TOF-TOF target plates using α-cyano-hydroxy-cinnamic acid as matrix. MS spectra were acquired using a Reflex III MALDI-TOF (Bruker Daltonics, Bremen, Germany); MS/MS spectra were acquired by using a 4700 MALDI-TOF-TOF (Applied Biosystem, Foster City, CA) and the 6 most abundant precursor ions were selected for fragmentation. MS and combined MS-MS/MS data were submitted to the MASCOT database search engine with the following searching parameters: SwissProt 57.3 protein database; carbamidomethylation as variable modification and oxidation of methionine and carbamylation as fixed modifications; one missed cleavage, mass tolerance of 0.1 Da for the peptide mass values, 0.1 Da for the ion mass values.

### N2a cell culture and toxicity assay

The murine neuroblastoma line N2a is frequently used when a cell line is needed which recapitulate neuronal features and it represents one of the common models for amyloid toxicity. N2a cells were grown in DMEM containing 4.5 g/l glucose (BE12-614F, Lonza, Basel, Switzerland) supplemented with 1×10^4^ U/ml penicillin/streptomycin (Gibco, Carlsband, CA), 2 mM L-glutamine (Gibco, Carlsband, CA) and 10% foetal calf serum (FCS, Lonza, Basel, Switzerland and heated at 56°C for 45 minutes to inactivate complement components) at 37°C, in 5% CO_2_. For toxicity assays confluent cells were trypsinized, diluted in DMEM containing 1% FCS to minimize cell growth and plated at the concentration of 5×10^4^ cells/ml in transparent, flat bottom 96-multiwell plates (Iwaky, Barloworld Scientific, UK). Cells were treated 4 hours after plating by adding 10 µl of peptide solution to the culture medium. Cell viability was determined 72 hours after treatment using a MTT reduction assay (Sigma-Aldrich, St. Louis MO), according to the manufacturer's directions.

## Supporting Information

Figure S1 Aβ 1–42 untagged and EDANS-tagged peptides were aged as previously described [7] to obtain SDS-stable, A11 positive aggregates (Figure S2B). The oligomeric morphology was evaluated by AFM. Peptide preparations showed a large number of rounded particles covering almost completely the mica surface with a mean height 3.1±0.1 nm (out of 250 randomly selected objects) (Figure S1A and S1B). CD spectra for both tagged and untagged oligomers showed the typical features of β-sheet secondary structure with a negative peak at 215 nm and a positive value around 195 nm (Figure S1C). Tagged and untagged oligomers were analyzed for their ability to impair N2a cell viability upon 72 hours treatment (concentration range from 0.5 nM to 5 µM). Decrease in the cellular reduction of MTT was considered a marker of cell viability. Aβ 1–42 untagged-oligomers were highly active, being toxic even at a concentration of 5 nM; EDANS-tagged oligomers caused a dose-related impairment of N2a cells viability comparable with Aβ 1–42 untagged oligomers (Figure S1D; mean and SD, N = 3 or more different experiments, 4 replicates each). Both Aβ 1–42 untagged and EDANS-tagged oligomers were able to produce amyloid fibrils upon incubation as demonstrated by EM after negative staining with uranyl acetate (Figure S1E–F).
(TIF)

Figure S2 An Aβ 1–42 batch of lyophilized peptide was disaggregated as previously described to obtain a preparation closed to the monomeric form of the peptide [7]. The Aβ 1–42 sample obtained was completely free of SDS-resistant species (Figure S2A) and was not recognized by A11 anti-oligomers antibody (Figure S2B). This sample contained very few and dispersed particles (average height: 0.56±0.03 nm, mean ± S.E.) as verified by AFM, however most of the mica surface was free of structured material (Figure S2C). In the ThT test, the sample at time zero did not produce a signal higher than the background, suggesting that it did not contain pre-formed species; moreover the signal growth was very slow in the first 24 hours of incubation (data not shown). A batch of lyophilized Aβ 1–40 was disaggregated using the same procedure. The disaggregated Aβ 1–40 peptide obtained did not possess SDS stable seeds and it was not recognized by the A11 antibody (data not shown), it was also analyzed by AFM which showed that no particle structures were visible (Figure S2C). The sample was therefore considered completely free of peptide aggregates. In order to confirm the Aβ 1–40 enrichment in monomers, nuclear magnetic resonance (NMR) was employed. Diffusion Order SpectroscopY (DOSY) experiments were done to measure the diffusion coefficient of the species in solution. The expected diffusion coefficients for solutions composed exclusively by monomers or dimers or trimers were calculated from standard curves. The diffusion coefficient for the solution of reversed Aβ 1–40 peptide was between that expected for a solution composed exclusively of monomers and for a solution containing only dimers (Table S1). Disaggregated Aβ 1–40 peptide was enriched in oligomers by incubation in 100 mM TRIS-HCl buffer pH 7.4 for 30 days at 37°C with 5 minutes shaking at 1000 rpm every 30 minutes. The aged Aβ 1–40 sample was analyzed by electron and atomic force microscopy. Most of the peptide was aggregated in round particles with a tendency to cluster and collapse in dense cores (data not shown). Finally Aβ 1–42 and Aβ 1–40 fibrils were obtained by incubating the monomeric peptides under acid conditions at 37°C for 40 or 10 days respectively. Electron micrographs of these last samples confirmed a substantial amount of mature fibrils (Figure S2D) which were long, narrow and clustered in bunches.
(TIF)

Table S1 Diffusion coefficients of disaggregated Aβ 1–40 peptide in solution as determined by STD-NMR. Expected (as standard curves) and obtained diffusion (m^2^/sec) for Aβ 1–40 peptide at different temperatures (5, 25 or 37°C). Nuclear magnetic resonance (NMR) experiments were performed using a Varian 400-MHz Mercury (Varian, Palo Alto, CA) equipped with a *z*-axis gradient coil. Pulse field gradient NMR diffusion measurements give molecular size through the measurement of diffusion coefficients. ^1^H spectra were acquired with 128, 160, 256 or 512 transients and 2 s recycle delay. Diffusion experiments were performed employing an array of 20 or 30 spectra for each experiment (128, 256 or 512 transients each, with a 1 or 2 s recycle delay) varying the gradient strength from 3.33 to 19.4 G/cm^2^. The lengths of and delays between the gradient pulses were optimized depending on the experimental conditions and ranged between 0.002 and 0.005 s and 0.2–0.7 s, respectively. Data were fitted and diffusion coefficients determined with the *Dosytoolbox* software. (http://personalpages.manchester.ac.uk/staff/mathias.nilsson/software.htm)
(DOC)

Supporting Information S1 (DOC)
