# Supplementary figures and images for: The Molecular Assembly of Amyloid Aβ Controls Its Neurotoxicity and Binding to Cellular Proteins
Source: PLoS One. 2011 Sep 23;6(9):e24909. doi: 10.1371/journal.pone.0024909 (PMC3179491; doi:10.1371/journal.pone.0024909)

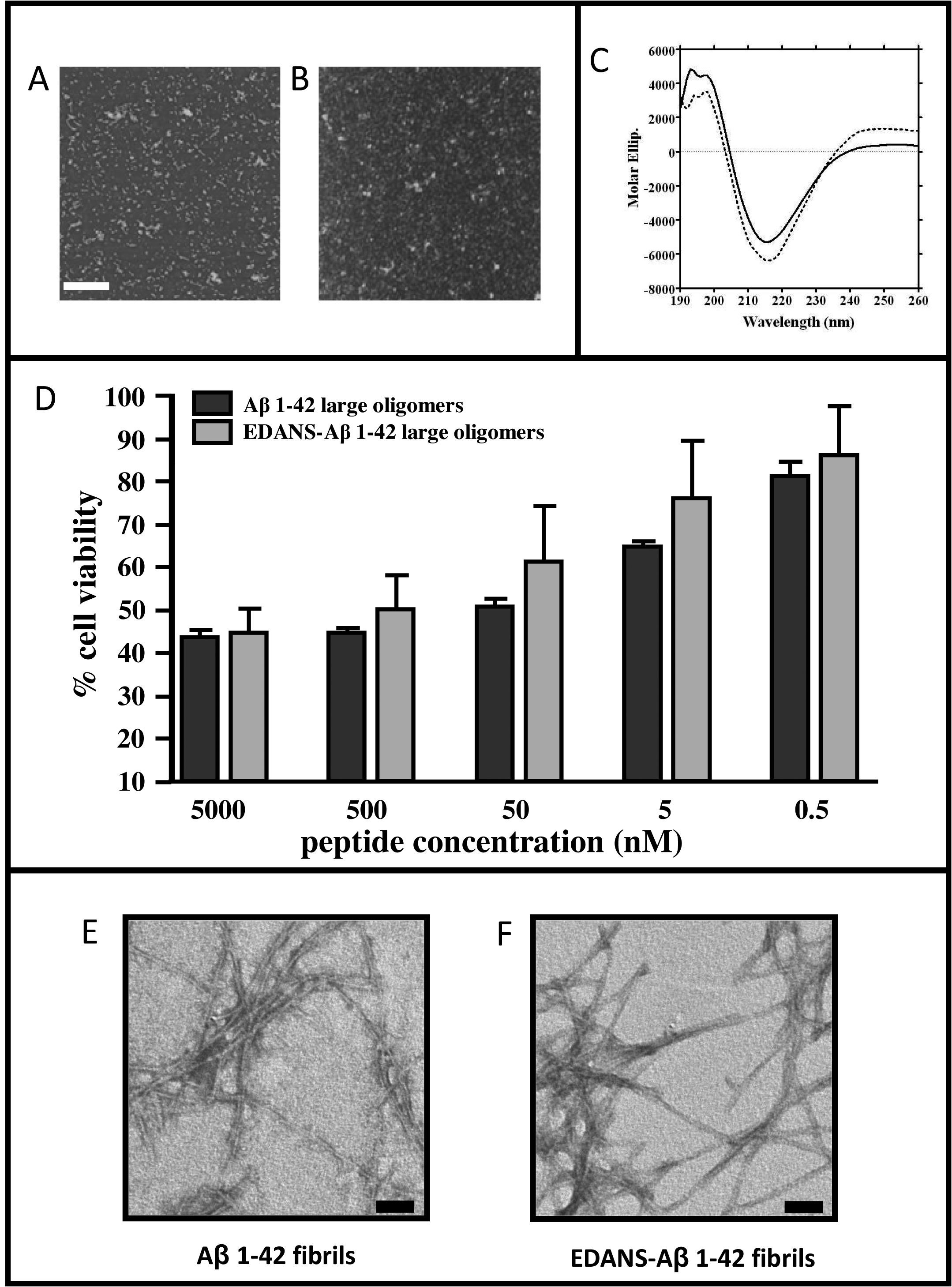

Supplement: Figure S1 — Aβ 1–42 untagged and EDANS-tagged peptides were aged as previously described [7] to obtain SDS-stable, A11 positive aggregates (Figure S2B). The oligomeric morphology was evaluated by AFM. Peptide preparations showed a large number of rounded particles covering almost completely the mica surface with a mean height 3.1±0.1 nm (out of 250 randomly selected objects) (Figure S1A and S1B). CD spectra for both tagged and untagged oligomers showed the typical features of β-sheet secondary structure with a negative peak at 215 nm and a positive value around 195 nm (Figure S1C). Tagged and untagged oligomers were analyzed for their ability to impair N2a cell viability upon 72 hours treatment (concentration range from 0.5 nM to 5 µM). Decrease in the cellular reduction of MTT was considered a marker of cell viability. Aβ 1–42 untagged-oligomers were highly active, being toxic even at a concentration of 5 nM; EDANS-tagged oligomers caused a dose-related impairment of N2a cells viability comparable with Aβ 1–42 untagged oligomers (Figure S1D; mean and SD, N = 3 or more different experiments, 4 replicates each). Both Aβ 1–42 untagged and EDANS-tagged oligomers were able to produce amyloid fibrils upon incubation as demonstrated by EM after negative staining with uranyl acetate (Figure S1E–F). (TIF) [file pone.0024909.s001.tif]

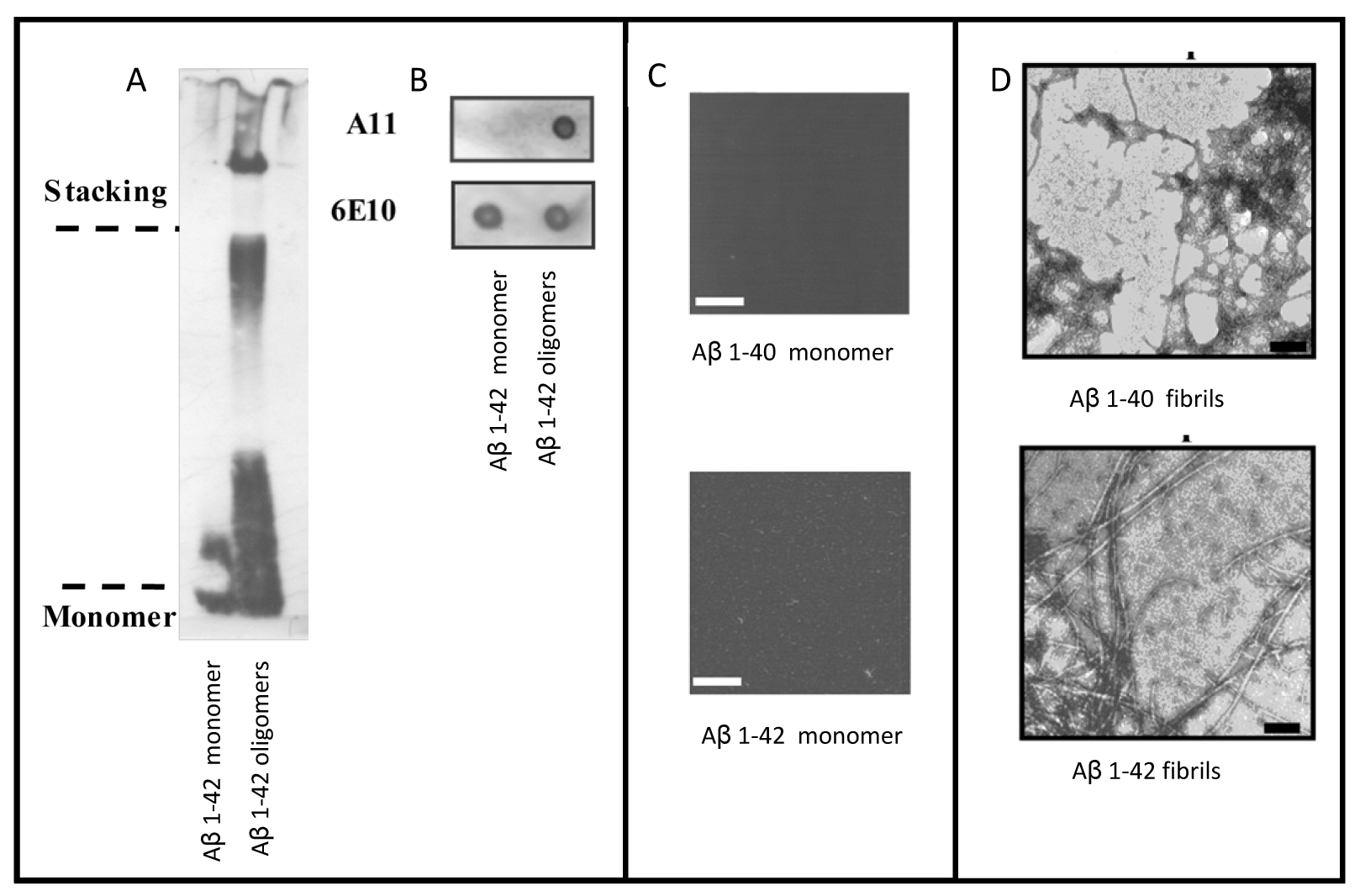

Supplement: Figure S2 — An Aβ 1–42 batch of lyophilized peptide was disaggregated as previously described to obtain a preparation closed to the monomeric form of the peptide [7]. The Aβ 1–42 sample obtained was completely free of SDS-resistant species (Figure S2A) and was not recognized by A11 anti-oligomers antibody (Figure S2B). This sample contained very few and dispersed particles (average height: 0.56±0.03 nm, mean ± S.E.) as verified by AFM, however most of the mica surface was free of structured material (Figure S2C). In the ThT test, the sample at time zero did not produce a signal higher than the background, suggesting that it did not contain pre-formed species; moreover the signal growth was very slow in the first 24 hours of incubation (data not shown). A batch of lyophilized Aβ 1–40 was disaggregated using the same procedure. The disaggregated Aβ 1–40 peptide obtained did not possess SDS stable seeds and it was not recognized by the A11 antibody (data not shown), it was also analyzed by AFM which showed that no particle structures were visible (Figure S2C). The sample was therefore considered completely free of peptide aggregates. In order to confirm the Aβ 1–40 enrichment in monomers, nuclear magnetic resonance (NMR) was employed. Diffusion Order SpectroscopY (DOSY) experiments were done to measure the diffusion coefficient of the species in solution. The expected diffusion coefficients for solutions composed exclusively by monomers or dimers or trimers were calculated from standard curves. The diffusion coefficient for the solution of reversed Aβ 1–40 peptide was between that expected for a solution composed exclusively of monomers and for a solution containing only dimers (Table S1). Disaggregated Aβ 1–40 peptide was enriched in oligomers by incubation in 100 mM TRIS-HCl buffer pH 7.4 for 30 days at 37°C with 5 minutes shaking at 1000 rpm every 30 minutes. The aged Aβ 1–40 sample was analyzed by electron and atomic force microscopy. Most of the peptide was aggregated in roun [file pone.0024909.s002.tif]
