# Supplementary material for: The Molecular Assembly of Amyloid Aβ Controls Its Neurotoxicity and Binding to Cellular Proteins
Source: PLoS One. 2011 Sep 23;6(9):e24909. doi: 10.1371/journal.pone.0024909 (PMC3179491; doi:10.1371/journal.pone.0024909)
Supplement: Table S1 — Diffusion coefficients of disaggregated Aβ 1–40 peptide in solution as determined by STD-NMR. Expected (as standard curves) and obtained diffusion (m2/sec) for Aβ 1–40 peptide at different temperatures (5, 25 or 37°C). Nuclear magnetic resonance (NMR) experiments were performed using a Varian 400-MHz Mercury (Varian, Palo Alto, CA) equipped with a z-axis gradient coil. Pulse field gradient NMR diffusion measurements give molecular size through the measurement of diffusion coefficients. 1H spectra were acquired with 128, 160, 256 or 512 transients and 2 s recycle delay. Diffusion experiments were performed employing an array of 20 or 30 spectra for each experiment (128, 256 or 512 transients each, with a 1 or 2 s recycle delay) varying the gradient strength from 3.33 to 19.4 G/cm2. The lengths of and delays between the gradient pulses were optimized depending on the experimental conditions and ranged between 0.002 and 0.005 s and 0.2–0.7 s, respectively. Data were fitted and diffusion coefficients determined with the Dosytoolbox software. (http://personalpages.manchester.ac.uk/staff/mathias.nilsson/software.htm) (DOC) [file pone.0024909.s003.doc]

| pH | T (°C) | Expected diff. coeff. for the ***monomer*** | Expected diff. coeff. for the ***dimer*** | Expected diff. coeff. for the ***trimer*** | ***Observed*** diff. coeff |
| --- | --- | --- | --- | --- | --- |
| 7.4 | 5 | 0.845 x10^-10^ | 0.696 x x10^-10^ | 0.622 x x10^-10^ | 0.745 x x10^-10^ |
| 7.4 | 25 | 1.626 x10^-10^ | 1.317 x x10^-10^ | 1.165 x x10^-10^ | 1.378 x x10^-10^ |
| 7.4 | 37 | 2.841 x x10^-10^ | 2.127 x x10^-10^ | 1.796 x x10^-10^ | 1.862 x x10^-10^ |

Diffusion coefficients in m2/sec; uncertainly less than 1%
