# Supplementary material for: The Molecular Assembly of Amyloid Aβ Controls Its Neurotoxicity and Binding to Cellular Proteins
Source: PLoS One. 2011 Sep 23;6(9):e24909. doi: 10.1371/journal.pone.0024909 (PMC3179491; doi:10.1371/journal.pone.0024909)
Supplement: Supporting Information S1 — (DOC) [file pone.0024909.s004.doc]

**Information S1**

*Peptides SDS-PAGE and Western Blot assays-* Peptide solutions were diluted 1:1 with the loading buffer containing 12% (w/v) sodium dodecyl sulphate (SDS) and immediately denatured at 100°C for 5 minutes. Samples were analyzed using Tris-Tricine SDS-PAGE (1.5 mm thick, 10-17.5% handmade gradient gels), followed by Western Blot analysis with primary 6E10 antibody (1:5000, Signet Laboratories, Dedham, MA), anti-mouse HRP conjugated secondary antibody (1:2000, DAKO, Carpinteria, CA) and an ECL detection kit (GE Healthcare Easton Turnpike, CT).

*A11 Dot Blot assay-* For Dot Blot assays, 4 µg of Aβ peptides were spotted on nitrocellulose membranes (0.2 μm filter paper, Whatman, GE Healthcare, Easton Turnpike, CT). After air drying membranes were blocked overnight in 10 mM Tris-HCl, pH 7.4, 100 mM NaCl, 0.1% Tween-20 (TBST) supplemented with 5% non-fat milk (Nestlé, Vevey, Switzerland). Membranes were washed in TBST and incubated sequentially using A11 anti-oligomer antibody (1:800; BioSource, Carlsband, CA), anti-rabbit peroxidase-conjugated secondary antibody (1:5000, Sigma-Aldrich, St. Louis MO) and an ECL detection kit (GE Healthcare Easton Turnpike, CT).

*Atomic Force Microscopy (AFM)-* Peptide samples were diluted with 10 mM HCl and 60 µl of sample was immediately spotted onto a freshly cleaved muscovite mica disk (Veeco/Digital Instruments, Mannheim, Germany). The disk was then washed with milliQ water and dried under a gentle nitrogen stream. Samples were mounted onto a Multimode AFM with a NanoScope V system (Veeco/Digital Instruments, Mannheim, Germany) operating in Tapping Mode using standard phosphorus-doped silicon probes (Veeco, Mannheim, Germany).

*Electron Microscopy (EM)-* Ten µl of peptide solution was dropped onto copper formvar carbon-coated 400-mesh EM grids (AGAR Scientific, Stansted, UK). Samples were then stained with a saturated solution of uranyl acetate in water for 5 minutes. EM was done with a Zeiss Libra 120 transmission electron microscope operating at 120 kV equipped with a Proscan Slow Scan CCD camera (Carl Zeiss SMT, Oberkochen, Germany).

*CD spectra of Aβ1-42 and EDANS-Aβ 1-42.* Aβ1-42 and EDANS-Aβ1-42 oligomers were dissolved 40 µM in 50 mM phosphate buffer (pH 7.4). Far-UV CD spectra were acquired with a band width of 1.0 nm and a resolution of 0.1 nm (J-815 CD-Spectrometer). Generally, a sensitivity of 100 mdeg, a response of 16 sec, a scan speed of 20 nm/min and three scan accumulations were used. CD spectra were expressed as mean of molar ellipticity. All measurements were performed at 37 °C
